# Calories and Fires

**Published:** 1912-02

**Authors:** 


### Calories and Fires.

It is a far cry from dietetics to the prevention of loss of life and property by conflagrations but the recent Equitable Building fire in New York and the destruction of fire-proof buildings with their contents in Baltimore a few years ago, lead us to suggest that a new problem has developed for firemen, insurance companies, building engineers and others especially interested, which, in our humble opinion, can be satisfactorily solved only by a study of caloric values, quite analogous to that which the dietetician applies to foods.

As the result of empiricism, many centuries old, man is in possession of information as to combustible or fire proof qualities of various substances used in building. As a result of more recent empiricism, it has been demonstrated that incombustible material subjected to heat, will crumble, crack, warp or otherwise succumb, and that combustible material inclosed in the nearest approach to non-conductive material will be destroyed and that these disastrous results may be due to combustion of parts of a building of very minor proportion to the whole, or of ordinary contents, or of surrounding structures.

In theory, and to some extent in practice, the contents of any room of a fire proof building may burn up without endangering adjoining apartments and the burning of a non-fire-proof structure near a fire proof one, should not damage the latter to any great degree. That there are exceptions to these rules, has been learned by bitter experience. It has also been learned that, within reasonable limits of size and height—say two or three stories—a substantially built structure composed almost entirely of wood is about as safe for the inmates, about as likely to be saved by fire apparatus of modern type, and involves about as little liability of loss of valuables inclosed in safes and vaults, as one composed almost entirely of fire proof material.

The special lesson of certain fires in New York and Philadelphia, has been to emphasize the fact that, so far as safety of inmates is concerned, fire proof construction is valueless if the contents are highly inflammable; while the special lesson of the Baltimore fire was that a building almost perfectly fire proof, and free from special danger from contents was not a protection to contents, human or otherwise and could not even maintain its own skeletal integrity, if subjected to extrinsic heat from large fires occurring in combustible and partially fire proof surrounding structures. In fact, the tendency to build fire proof structures to a great height, makes them especially dangerous to occupants, contents, fire fighters and wreckers, if they are subjected to a certain heat strain.

These lessons show that men professionally interested in fires and professionally responsible for their prophylaxis and treatment have reached a stage which the men professionally interested in disease and professionally responsible for its prophylaxis and treatment, reached some years ago—namely, the stage at which qualitative methods have proved inadequate and must give place to scientific quantitative study.

In other words, it must be known how much combustible material may safely be worked into a building, as doors, wainscoating, mouldings, sashes, etc.; how much combustible furniture and goods may be placed in a room without endangering the life of its occupants and the integrity of other rooms in the same building; how many and how large buildings of freely combustible or slow burning construction may be placed in a given area, without endangering strictly fire proof buildings;—and many more details of like nature must be definitely known.

This involves a scientific study of caloric potentialities of different kinds of material, weighing, study of heat convection under different circumstances.

